# An explorative cross-sectional study examining self-reported health and nutritional status of disadvantaged people using food banks in Germany

**DOI:** 10.1186/s12939-015-0276-6

**Published:** 2015-11-24

**Authors:** Julia Depa, Carolin Hilzendegen, Peter Tinnemann, Nanette Stroebele-Benschop

**Affiliations:** Institute of Nutritional Medicine, Department of Nutritional Psychology, University of Hohenheim, Fruwirthstraße 12, 70599 Stuttgart, Germany; Institute for Social Medicine, Epidemiology and Health Economics, Charité University Medical Center, Luisenstr. 57, 10117 Berlin, Germany

**Keywords:** Food bank, Health, Nutrition, Low socioeconomic status

## Abstract

**Background:**

Even in high-income countries some population groups depend on food banks to support their food intake. We aimed to explore and compare health and nutritional status of food bank clients (Tafel e.V.) in different cities in Germany.

**Methods:**

In a cross-sectional study, self-reported health and nutritional status of food bank clients living in three cities (Berlin - capital, Ludwigsburg- affluent city, Fulda - small town) which differ in size, available income and poverty rate, were assessed and compared to survey variables of the low socioeconomic status population of national surveys (DEGS and GEDA).

**Results:**

Across cities, food bank clients (*N* = 276, response rate of 21.5 %) did not differ in main socio-demographic characteristics (age, nationality, education, professional qualification, household income). Smoking, having at least one chronic illness, estimating their own health status as moderate to poor and low consumption of fruits and vegetables were common characteristics. Comparing selected variables with the low socioeconomic status population of DEGS and GEDA, differences were found for a higher prevalence of diabetes among food bank clients and a worse self-reported health status. Considerably lower fruit consumption and lower hypertension prevalence among female and lower overweight rates among male food bank clients were found.

**Conclusions:**

Although people using food banks vary in socio-demographic background, no differences for main demographics across the cities were found. In addition, the study suggests that for some health- and nutrition-related variables, national surveys in Germany might underestimate socioeconomic differences.

## Background

The socioeconomic status (SES) influences people’s morbidity, premature death and life expectancy within a country ([12]; [10]). People with lower SES suffer more often from several non-communicable diseases such as diabetes ([9]; [25]), cardiovascular disease or some forms of cancer [9, 12, 16]; [25]) than people with higher SES. They also complain more often about poor self-reported health status [9, 22]. Behavior-related risk factors such as high BMI [9, 33], physical inactivity [9, 23], smoking [11, 23] as well as low consumption of fruits and vegetables and a diet high in fat and sugar content ([5]; [14]; [18]; [25]) are also more prevalent among low SES groups.

Health inequality data is commonly obtained with nationwide surveys, although these types of studies are believed to often underrepresent low-income households [37], partly caused by recruitment and survey methods used (e.g. standardized and non-cultural specific cover letters and survey instruments), study-related constraints (e.g. providing informed consent, which was reported as relinquishing rather than protecting rights) and general minority groups’ low tendency to participate in research stirred by mistrust in government agencies [4, 39]. Furthermore lower literacy, numeracy and language skills of low-income households make it difficult for the participants to complete dietary records. Elderlies cooperation rates can also be limited because of diverse physical (poor eyesight, impaired hearing, being chair or bed-ridden) and health problems (dementia) [8]. In the second German national nutrition survey (NVS II), migrants, residents in institutions, persons without a permanent home, children under the age of 14, families with children, and elderly living on their own are clearly underrepresented [24]. This potentially leads to an underrepresentation of poor people and population groups at a higher risk of nutritional poverty [13]. A similar problem is the case for the DEGS (German health interview and examination survey for adults) [26] and GEDA (German Health Update) [25] because of the above described limitations.

Hence, it is speculated that disparities between socioeconomic groups in regards to health and health-related behavior could be higher than found by conducted representative German surveys.

In high-income countries such as the United States, Canada, all of Europe including Germany, some household income is so low, that they depend on food banks. Food banks, mostly organized as nonprofit-organizations, collect food from farms, manufacturers, distributors, retail stores, consumers, as well as other sources and distribute the donations for free or for a small fee to people in need [34]. In Germany, it is estimated that 1,5 Million people visit local food banks on a regular basis. Fifty-three percent of the food bank clients are commonly recipients of unemployment benefit (primarily the so called ALG II) or basic security. Moreover, 17 % of the food bank clients are seniors and 25 % are migrants or late repatriates [3]. In-depth research on this population group could provide relevant information about health-related behavior in people with low SES (operationalized by a low income), a particular population group that appears to be missing in representative survey data. Until today, only limited research results in regards to health and eating behavior of this group in Europe are available [15, 20, 36].

The aim of this study was to examine the health and nutrition status of people using food banks in three different types of cities in Germany, to identify differences among those and to compare available health- and nutrition-related variables of this sample with the as low defined SES population in the DEGS and GEDA.

## Methods

### Data collection and participants

A cross-sectional study was conducted at three food bank locations in Germany: Berlin (survey period: October and November 2009), Ludwigsburg (survey period: May and June 2013) and Fulda (survey period: April 2014). The cities differ in size, available income per person and poverty rate. The available income per person (and the poverty rate) are lowest in Berlin, followed by Fulda and then Ludwigsburg ([27]; [30]; [31]).

In Berlin, the capital of Germany, three food redistribution points, located in the districts Reinickendorf, Marzahn and Kreuzberg were visited for data collection. These food redistribution points offer food on a weekly basis. Every distribution point was visited for data collection four times. In Fulda (a small town in Hesse with about 65.000 residents), the location opens three times per week and data collection was conducted on those three days for two weeks. In Ludwigsburg (a rather affluent town in Baden-Württemberg, north of Stuttgart, with about 91.000 residents), the visited food bank store has daily opening hours. Therefore, recruitment took place on three different days for three hours each.

At all locations, people were asked whether they would be willing to fill out a short survey about their health and nutrition status while they were waiting in line to enter the store. If Tafel clients agreed, it was pointed out that participation is voluntary and no information allowing identification of individuals participating in the survey was collected. Food bank clients who had difficulties reading or understanding the questions were offered help filling in the questionnaire. Inclusion criteria were age 18 years or older and the permission to use the food bank at the surveyed location. This study was exempt from ethical approval by the Charité University Medical Center (for Berlin) and the University of Hohenheim (for Ludwigsburg and Fulda) due to its study procedures. Written approval was obtained from Tafel officials at each location.

### Measurement tools

The self-developed questionnaire integrated questions of the DEGS, adapted to the study population [26]. The DEGS is the first nationwide representative longitudinal survey on the health status of Germany’s adult population [26].

Participants were asked to answer questions regarding age, sex, nationality, country of birth, school education, highest professional qualification, source of income, household income, household size, household composition and marital status. Questions regarding health behavior included self-reported weight and height, self-related health status, smoking behavior, alcohol consumption and different chronic illnesses. The classification of overweight and obesity is based on the WHO classifications [38]. Nutrition status was assessed using questions regarding meal frequencies, food purchasing behavior, food costs and the consumption of different food groups.

The used questionnaire differed only in the selection of questions across the three locations since they were administered by different universities and staff members. In Ludwigsburg, the participants were not asked for nationality, country of birth, household income, alcohol consumption, smoking, chronic illnesses and consumption of fresh cooked food upon request of the food bank officials. In Berlin, the participants were not asked to report meat, meat products and fish consumption. In Ludwigsburg, the questionnaire differed also slightly in terms of the wording of the questions about the consumption of different food groups. For instance, it was not distinguished between raw and cooked vegetables. In addition, in Ludwigsburg, the following question was used: “How often do you usually consume fruits?” compared to the question “How often did you consume fruits in the last four weeks?” in Berlin and Fulda. Possible answers for this question in Ludwigsburg were”daily”, “weekly”, “monthly” and “never” and in Berlin and Fulda “daily”,”often (more than once per week)”, “irregular (less than once per week)”, “seldom (less than once per month)”and “never”.

Besides in German, the questionnaire was available in Turkish at the Berlin location, in Turkish, Russian and Croatian at the Ludwigsburg location and in Turkish and Russian at the Fulda location. The languages were chosen based on information from the corresponding food bank officials in regards to clients’ nationality and on researchers' capability of a foreign language.

### Statistical methods

Differences between groups were tested for ordinal scaled and not normally distributed variables with the Mann–Whitney or the Kruskal Wallis test and for nominal scaled or dichotomous variables with the chi squared test. Given that age but not BMI was normally distributed, analysis of one-way analyses of variance were conducted. Criterion for statistical significance was set at *p* < .05. The questionnaires were analysed using SPSS Version 22*.* Adjusted analyses were conducted for sex based on the distribution of the German population [32].

The answer options “irregular (less than once per week)” and “seldom (less than once per month)” regarding the consumption of different food groups from Berlin and Ludwigsburg were summarized into the answer category “monthly” to compare it with data from Ludwigsburg.

Descriptive statistics were used to compare selected variables with the low SES population of the DEGS and GEDA. In the DEGS, low SES was operationalized by an index considering occupation, education and income [9]. In the GEDA, low SES was defined by a low education. Food bank clients in this study were categorized as having a low SES because of the very low income. For the comparison of BMI, self-reported health status, hypertension and diabetes, published data of the DEGS, and for the daily fruit consumption, published data of the GEDA were used.

## Results

A total of 276 questionnaires – 94 from Berlin, 64 from Ludwigsburg and 114 from Fulda – were analysed. The overall response rate was 21.5 % (Berlin 22.7 %, Ludwigsburg 21.3 %, Fulda 20.1 %). Since adjusting for sex did not reveal different results except for the prevalence of diabetes, results are reported without adjusting for sex.

### Baseline characteristics

Table 1 presents the baseline characteristics for all three food bank locations. No significant differences between the locations were found for age, nationality, school education, highest professional qualification, source of income and household income. Mean age of the sample population was 47.7 years (±14.4 years) and the median was 47.0 years. Primary education was the most frequent highest professional qualification, whereby at least over one-quarter (25.9 %) of the respondents had achieved university entrance schooling level and more than one-quarter (28.9 %) had a secondary education. Most participants (43.1 %) received unemployment benefits (ALG II) and 23.7 % received a pension. Sixty-seven percent of the food bank clients had a household income of below 750€ per month.

**Table 1:** Characteristics of food bank clients at all three locations (*n* = 276)

Socio-demographic variables	B (*n* = 94)	LB (*n* = 64)	FD (*n* = 114)	Total (*n* = 276)	*p*-value
Age [Mean (SD), min-max, median]	47.0 (±14.7)	49.1 (±13.8)	47.3 (±14.4)	47.7 (±14.4)	0.655
	19–83, 50.0	25–76, 47.5	21–84, 45.0	19–84, 47.0	
Sex [%]					0.007
female	54.1	78.1	60.5	62.3	
male	45.9	21.9	39.5	37.7	
Nationality [%] ^a)^					0.074
Germany	90.7		82.1	86.1	
other	9.3		17.9	13.9	
Country of birth [%] ^a)^					0.000
Germany	86.5		53.2	68.6	
other	13.5		46.8	31.4	
School education [%]					0.326
university entrance level	21.9	37.1	23.2	25.9	
secondary education	35.4	22.6	26.8	28.9	
primary education	35.4	32.3	44.6	38.5	
none/ not yet	7.3	8.1	5.4	6.7	
Highest professional qualification [%]					0.986
university	19.1	23.3	18.8	20.0	
apprenticeship	52.1	43.3	53.5	50.6	
still making apprenticeship	5.3	3.3	2.0	3.5	
no work education	23.4	30.0	25.7	25.9	
Source of income [%]					0.129
ALG I (1^st^ year of unemployment)	4.1	6.5	3.5	4.4	
ALG II (after 1^st^ year of unemployment pension)	52.0	40.2	36.8	43.1	
other (e.g. BaföG, fulltime job, part-time job,	20.4	30.6	22.8	23.7	
apprenticeship)	23.5	22.6	36.8	28.8	
Household income [%] ^a)^					0.120
under 750€	71.7		63.3	67.0	
750–950€	16.5		11.0	13.6	
950–1200€	6.2		12.8	9.7	
1200–1400€	3.1		10.1	6.8	
Over 1400€	3.1		2.8	2.9	
Single parent [%] ^a)^	17.0		33.8	24.4	0.012
Single household [%]	59.2	29.7	27.9	39.8	0.000
Household size [Mean (SD), min-max]	1.7 (±1.1), 1–7	2.8 (±1.7), 1–9	2.5 (±1.4), 1–6	2.3 (±1.5), 1–9	0.000
Marital status [%]					0.000
single	39.2	9.4	27.2	27.3	
married	14.4	51.6	40.4	33.8	
divorced	43.3	29.7	28.9	34.2	
widow	3.1	9.4	2.4	4.7	

^a)^ for Ludwigsburg no data available

*B* = Berlin , *LB* = Ludwigsburg , *FD* = Fulda

Participants at the different locations differed significantly regarding country of birth. In Fulda, more participants indicated a different country of birth than in Berlin (46.8 % vs. 13.5 %, *p* = 0.000). The majority of food bank clients (62.3 %) were females, whereas in Berlin fewer women (54.1 %) than at the other locations visited the distribution centers (60.5 % vs. 78.1 %, *p* = 0.007). Analysing all locations, single parent were 24.4 % of the respondents and 78 % of them were women. More single parents seemed to visit the locations in Fulda (33.8 %) than Berlin (17.0 %, *p* = 0.012). Therefore, more food bank clients appeared to live in single households in Berlin than in the other locations (59:2 % vs. 29.7 % and 27.9 %, *p* = 0.000) and considered themselves as single (39.2 % vs. 9.4 % and 27.2 %, *p* = 0.000) or divorced (43.3 % vs. 29.7 % and 28.9 %, *p* = 0.000). In general, household size in Berlin was smaller than in Fulda and Ludwigsburg (*p* = 0.000).

### Health and nutrition status

Table 2 shows the mean BMI of food bank clients with significant differences (*p* = 0.004) between locations. In Berlin, the BMI was lower (26.5) compared to Ludwigsburg (28.7) or Fulda (28.4). These differences were also seen in the distribution of the weight categories (*p* = 0.002). In Berlin, most food bank clients were normal weight (52.7 %) whereas in Ludwigsburg over a third of the respondents were obese (38.1 %) and in Fulda over a third were overweight (37.2 %). At these two locations, the majority of the food bank population had a BMI over 25.

**Table 2:** Health-related variables of food bank clients at all three locations (*n* = 276)

Health-related variables	B (*n* = 94)	LB (*n* = 64)	FD (*n* = 114)	Total (*n* = 276)	*p*-value
BMI [Mean (SD), min-max]	26.45 (7.3), 18–68	28.74 (6.0), 17–42	28.36 (7.6), 13–68	27.78 (7.2), 13–68	0.002
BMI categories [%]					0.004
underweight	1.1	1.6	1.8	1.5	
normalweight	52.7	28.6	32.7	38.7	
overweight	24.7	31.7	37.3	31.6	
obese	21.5	38.1	28.2	28.2	
Alcohol consumption ^a)^ [%]					0.011
never	41.2		55.4	48.8	
once a month or less	28.9		27.7	28.2	
2–4 times a month	13.4		12.5	12.9	
2–3 times a week	10.3		2.7	6.2	
4 times a week or more	6.2		1.8	3.8	
Smoker %	60.4		35.4	46.9	0.000
Chronic illness ^a)^	66.3		64.5	65.4	0.203
asthma	15.3		12.5	13.8	0.557
diabetes	6.1		13.5	10.0	0.076
hypertension	18.4		30.6	24.9	0.041
back pain	34.7		38.4	36.7	0.579
rheumatism	9.2		8.0	8.1	0.767
other	41.8		17.9	29.0	0.000
Self-reported health status ^a)^ [%]					0.700
very good	3.1		7.0	5.2	
good	33.0		21.1	26.5	
moderate	42.3		52.6	47.9	
bad	15.5		14.9	15.2	
very bad	6.2		4.4	5.2	

^a)^ for Ludwigsburg no data available

*B* = Berlin , *LB* = Ludwigsburg , *FD* = Fulda

Furthermore, over two-third (76 %) of the respondents drank alcohol once a month or not at all. In Berlin, alcohol consumption was significantly higher than in Fulda (*p* = 0.011). Smokers accounted for 46.9 % of the respondents, whereby the amount was considerably higher in Berlin than in Fulda (60.4 % vs 35.5 %, *p* = 0.000). More than every second respondent (65.4 %) suffered from at least one chronic illness (asthma, diabetes mellitus, hypertension, back pain, rheumatism). Back pain (36.7 %) and hypertension (24.9 %) were indicated most. Comparing the presence of chronic illnesses between locations, no significant differences were found, except for Fulda where more respondents suffered from hypertension than in Berlin (30.6 % vs 18.4 %, *p* = 0.041).

Most respondents (47.9 %) estimated their health status as moderate. Between the three locations no significant differences (*p* = 0.7) existed regarding the subjective estimate of their health status.

Table 3 shows the nutrition-related behavior including the consumption of different food groups and the purchase of additional food in general stores. Most of the sample population indicated that fruits were eaten daily (50 %). Raw and cooked vegetables, meat and meat products were most frequently eaten weekly (33.7 %, 28.8 % and 31.2 %, respectively). In regards to location differences in the consumption of fruits and vegetables, more respondents from Ludwigsburg appeared to consume fruits (*p* = 0.000) and vegetables (*p* = 0.000) – raw and cooked – on a daily basis than in Berlin or Fulda. Excluding Ludwigsburg showed that the consumption of fruits and raw vegetables differed between the remaining locations (*p* = 0.028 and *p* = 0.001, respectively), but not for cooked vegetables (*p* = 0.807). In Fulda, more food bank clients cooked daily with fresh products than in Berlin (54.5 % and 37.8 %, *p* = 0.009). No differences across all locations were found for the consumption of convenience products (*p* = 0.133).

**Table 3:** Nutrition-related behavior of food bank clients at the three locations (*n* = 276)

Nutrition-related behavior variables	B (*n* = 94)	LB (*n* = 64)	FD (*n* = 114)	Sample Population (*n* = 276)	*p*- value
Fruit consumption					0.000
Daily	38.1	71.4	48.2	50.0	
Weekly (more than once per week)	26.8	27.0	32.1	29.0	without LB: 0.028
Monthly (less than once per week)	32.0	1.6	18.8	19.5	
Never	3.1	0.0	0.9	1.5	
Consumption raw vegetables ^b)^					.000
Daily	20.6	66.7	26.5	33.7	
Weekly (more than once per week)	26.8	31.7	47.8	36.6	without LB: 0.001
Monthly (less than once per week)	48.5	1.6	24.8	27.8	
Never	4.1	0.0	0.09	1.8	
Consumption cooked vegetables ^b)^					.000
Daily	21.4	66.7	14.2	28.8	without LB: 0.807
Weekly (more than once per week)	43.9	31.7	54.9	45.6	
Monthly (less than once per week)	31.6	1.6	28.3	23.4	
Never	3.1	0.0	2.7	2.2	
Meat consumption ^c)^					0.567
Daily	/	28.6	32.7	31.2	
Weekly (more than once per week)		60.3	43.6	49.7	
Monthly (less than once per week)		7.9	20.9	16.2	
Never		3.2	2.7	2.9	
Consumption of convenience products					0.133
Daily	3.1	4.9	6.7	4.6	without LB: 0.039
Weekly (more than once per week)	9.3	26.2	7.6	12.5	
Monthly (less than once per week)	59.8	37.7	69.5	58.6	
Never	28.9	31.1	16.2	24.3	
Consumption freshly cooked ^a)^					0.009
Daily	37.8		54.5	46.7	
Weekly (more than once per week)	36.7		31.2	33.8	
Monthly (less than once per week)	24.5		13.4	18.6	
Never	1.0		0.9	1.0	
Buying additional food in stores ^a)^					
Fruit	52.0		51.8	51.9	0.971
Vegetables	52.0		50.0	51.0	0.768
Bread, baked goods	50.0		34.8	41.9	0.026
Pasta, rise, potatoes	70.4		79.5	75.2	0.129
Meat, meat products and fish	75.5		87.5	81.9	0.024
Milk, milk products	80.6		77.7	79.0	0.602
Convenience products	29.6		35.7	32.9	0.346
Sweets	48.0		49.1	48.6	0.868
Beverages	73.5		81.2	77.6	0.177

^a)^ for Ludwigsburg no data available, ^b)^ Ludwigsburg asked only for vegetables in general (no distinction between between raw and cooked), ^c)^ for Berlin no data available

*B* = Berlin , *LB* = Ludwigsburg , *FD* = Fulda

Although fruits and vegetables are the main products to purchase at the food banks, half of respondents indicated to buy additional fruits (51.9 %) and vegetables (51 %). More than two-third of the food bank clients also additionally purchased pasta, rice or potatoes (75.2 %), milk and milk products (79 %), beverages (77.6 %) as well as meat, meat products and fish (81.9 %). Convenience food was bought by 32.9 % of the respondents.

Adjusted for sex, health- and nutrition-related differences were not found between the cities, except in Fulda, more food bank clients suffered from diabetes than in Berlin (13.5 % vs. 6.1 %, *p* = 0.036).

### Comparison of selected variables with the low SES population using DEGS and GEDA data

As seen in Table 4, compared to the general population with a low SES as assessed in the DEGS, the amount of overweight persons (women 59.2 %, men 66.5 %) [17] was slightly higher among women using food banks (62.7 %) and noticeably lower among men (55.0 %). The amount of obese persons, however, was lower among food bank clients (women 28.4 %, men 28.0 %) than in the low SES population of the DEGS (women 28.3 %, men 36.2 %) [9].

**Table 4:** Comparison of selected health- and nutrition-related variables of the sample population with the low SES population of the DEGS (low SES operationalized by an index considering occupation, education and income) and the GEDA (low SES was operationalized by a low education)

Health status and eating behavior	food bank clients	low SES population (DEGS^a)^ and GEDA ^b)^)
BMI ≥25 ^a)^ [%]		
men	55.0	66.5
women	62.7	59.2
BMI ≥30 ^a)^ [%]		
men	28.0	28.8
women	28.4	36.2
Self-rated health status (moderate, bad and very bad) ^a)^ [%]		
men	67.4	43.5
women	68.8	36.7
Hypertension ^a)^ [%]		
men	28.1	32.3
women	22.5	37.1
Diabetes ^a)^ [%]		
men	14.6	6.2
women	6.7	3.0
Daily fruit consumption ^b)^ [%]		
men	39.8	43.5
women	56.2	62.4

^a)^ variables of the DEGS (German health interview and examination survey for adults)

^b)^ variables of the GEDA (German Health Update)

Hypertension was less prevalent among food bank clients for both sexes (men 28.1 %, women 22.5 %) than in the low SES population of the DEGS (men 32.3 %, women 37.1 %) [21]. Contrary, food bank clients of both sexes suffered twice as often from diabetes (men 14.6 %, women 6.7 %) than the low SES population of the DEGS (men 6.2 %, women 3.0 %) [7]. Additionally, the amount of food bank clients who rated their health status as moderate, bad and very bad was markedly higher (68.2 %) compared to the population with a low SES of the DEGS (women 43.5 %, men 36.7 %) [9]. Furthermore, considerably more food bank clients indicated to smoke compared to the general population of the DEGS (46.9 % and 29.7 %) [11] (data not shown in Table 4, since no data was available for the low SES group of the DEGS).

The daily consumption of fruits was higher in the low SES population of the GEDA (men 43.5 %, women 62.4 %) than among food bank clients (men 39.8 %, women 56.2 %) for both sexes. When only analysing food banks clients with a minimum education, fruit consumption among men was approximately the same and considerably lower among women (men 42.9 %, women 37.0 %) compared to the GEDA population with a low education.

## Discussion

The study presented the first description of self-reported health- and nutrition-related behavior of a sample of German food bank clients from different cities varying in size, available income per person and poverty rate (Berlin, Ludwigsburg and Fulda). Although the cities differed in size and demographics, the rather heterogeneous group of people using food banks were similar across the locations in regards to their socio-demographic characteristics such as age, nationality, school education, highest professional qualification, source of income and household income. Thus, it can be assumed that disadvantaged people in need of additional food can be found across all ages, gender, and level of education.

In general, being a single parent and being of older age is associated with a higher poverty risk [1, 6]. Our study results confirmed this partly. Compared to the general German population, the amount of single parents was higher among food bank clients (19.0 % vs. 24.4 %) [29]. Also, on average, food bank clients were older (43.9 vs. 47.7) [32] and the median (45.3 vs. 47.0) [28] was also higher. However, the amount of persons receiving pension payment was similar to the number of German pensioners (23.7 % vs 23.7 %) [2]. Furthermore, it is important to note that over half of the respondents had at least a secondary education or an apprenticeship. One quarter had a university entrance level education and 20 % had a university degree. This seems to indicate that a higher level of education is not necessarily protective of poverty [35].

When only looking at Berlin and Fulda, less than 50 % of the participants reported to eat fruits on a daily basis and not even a third consumed raw or cooked vegetables daily. Although fruits and vegetables are the main foods available at German food banks, about 50 % of the participants bought additional fresh produce at other shopping locations. Bread is also a main food product offered at German food banks but it was less often bought in addition. Looking at other food products such as milk or meat products, the percentage was even higher. Food banks such as Tafel e.V. aim to mainly provide additional fresh produce and are not meant to provide a sufficient provision of foods. Nevertheless, given its focus on fruits and vegetables it seems important to note that even their offered supply cannot cover the recommended daily consumption of fruits and vegetables.

Comparing available variables with data of the low SES group of the representative German surveys (DEGS and GEDA), the biggest differences were found for the higher prevalence of diabetes, lower self-reported health status among food bank clients and lower fruit consumption as well as fewer female food bank users in the BMI > 25 category. Furthermore, fewer men were considered overweight and fewer women were considered obese compared to the low SES population. However, the discrepancies in BMI should be considered with caution given the well-known bias when using self-reported height and weight measurements [19].

The health discrepancies, however, might be also caused by the above mentioned assessment difficulties [4, 8, 37, 39] that can lead to the underrepresentation of disadvantaged people in nationwide surveys. Besides, these findings go hand in hand with the general findings of the unequal distribution of disease and risk factors among low-income people ([5, 12, 16]; [14]; [9, 11, 22, 23]).

In Germany, nearly 1.5 Million people use food banks. Considering the response rate of 21.5 %, which is comparable with nationwide surveys in Germany (22.1 %) [25], conducting research in this particular setting seems feasible although different recruitment and assessment procedures need to be applied.

### Limitations

Several study limitations need to be mentioned. The use of self-reported questionnaires in general has several limitations such as response, recall or selection bias. Proneness to bias is particularly prevalent in self-reported height and weight [19]. In addition, the relatively high education level among our sample might have been caused by our study procedures. It is possible that food bank clients with lower educational status were hesitant in participating, a fact that has been repeatedly described [4, 24, 39]. Another possible selection bias could have occurred by using a Russian questionnaire in Fulda and having a Turkish speaking researcher in Berlin. Speaking the native language appears to promote participation and might have disproportionally increased the number of foreigners. However, looking at the data, this appears to be only the case in Fulda.

Another limitation is the difference in time of data collection and the use of a slightly different array of questions used across locations. In Berlin, data collection took place during the fall and about three years earlier (October and November, 2010) than in Ludwigsburg (May and June, 2013) or Fulda (April, 2014). Therefore, existing differences could also be explained by time.

Furthermore, the results of comparing food bank clients with the low SES population of DEGS and GEDA should be treated with caution. Data of the DEGS and the GEDA were obtained from published articles and not original databases and the differences in age distribution between the nationwide surveys and our study population might have played a role.

Overall, given the small sample size, our results need to be seen as explorative in nature and not generalizable for German people with low SES or for all food bank clients.

## Conclusions

Research on eating habits and health status of people with very low SES are rare in Germany and Europe in general and using food banks as a way to reach these people to introduce health promoting public health strategies is still in its infancy.

Considering the above described limitations, comparisons should also take place with the original database of the nationwide surveys and further analyses with a larger and more reliable sample of food bank clients will be necessary to confirm our results about the possible underestimated socioeconomic differences in health- and nutrition-related variables in nationwide surveys.

## References

[1] bpb (2014a) Ausgewählte Armutsgefährdungsquoten. In Prozent, 2011. http://www.bpb.de/nachschlagen/zahlen-und-fakten/soziale-situation-in-deutschland/61785/armutsgefaehrdung. Accessed 11 June 2015

[2] bpb (2014b) Rentner (RV). Anteil der Rentner an der Bevölkerung der Bundesländer, Rentner in absoluten Zahlen 2012. http://www.bpb.de/nachschlagen/zahlen-und-fakten/soziale-situation-in-deutschland/61848/rentner. Accessed 11 June 2015

[3] Bundesverband Deutsche Tafel e.V. (2015) Zahlen & Fakten. http://www.tafel.de/die-tafeln/zahlen-fakten.html. Accessed 11 June 2015

[4] Corbie-Smith GM (2004). Minority Recruitment and Participation in Health Research. Commentary. NC Med J.

[5] Darmon N, Drewnowski A (2008). Does social class predict diet quality?. Am J Clin Nutr.

[6] Goebel J, Grabka MM (2011). Zur Entwicklung der Altersarmut in Deutschland. DIW Wochenbericht.

[7] Heidemann C, Du Y, Schubert I, Rathmann W, Scheidt-Nave C (2013). Prevalence and temporal trend of known diabetes mellitus. Results of the German Health Interview Examination Survey for Adults (DEGS1). Bundesgesundheitsbl.

[8] Holmes B, Dick K, Nelson M (2008). A comparison of four dietary assessment methods in materially deprived households in England. Public Health Nutr.

[9] Lampert T, Kroll L, von der Lippe E, Müters S, Stolzenberg H (2013). Socioeconomic status and health: Results of the German Health Interview and Examination Survey for Adults (DEGS1). Bundesgesundheitsbl.

[10] Lampert T, Le K (2014). Soziale Unterschiede in der Mortalität und Lebenserwartung. GBE Kompakt 5 (2).

[11] Lampert T, von der Lippe E, Müters S (2013). Prevalence of smoking in the adult population of Germany: Results of the German Health Interview and Examination Survey for Adults (DEGS1). Bundesgesundheitsbl.

[12] Mackenbach JP, Stirbu I, Roskam AR, Schaap MM, Menvielle G, Leinsalu M (2008). Socioeconomic inequalities in health in 22 European countries. N Engl J Med.

[13] Max Rubner-Institut (2005) Nationale Verzehrsstudie II. Study design and organisation. http://www.was-esse-ich.de/index.php?id=46. Accessed 11 June 2015

[14] Max Rubner-Institut (2008). Nationale Verzehrsstudie II. Ergebnisbericht Teil 2: Die bundesweite Befragung zur Ernährung von Jugendlichen und Erwachsenen.

[15] Méjean C, Deschamps V, Bellin-Lestienne C, Oleko A, Darmon N, Serge H (2010). Associations of socioeconomic factors with inadequate dietary intake in food aid users in France (The ABENA study 2004–2005). Eur J Clin Nutr.

[16] Melchior M, Goldberg M, Krieger N, Kawachi I, Menvielle G, Zins M (2005). Occupational class, occupational mobility and cancer incidence among middle-aged men and women: a prospective study of the French GAZEL cohort. Cancer Causes Control.

[17] Mensink G, Schienkiewitz A, Haftenberger M, Lampert T, Ziese T, Scheidt-Nave C (2013). Overweight and obesity in Germany. Results of the German Health Interview and Examination Survey for Adults (DEGS1). Bundesgesundheitsbl.

[18] Mensink G, Truthmann J, Rabenberg M, Heidemann C, Haftenberger M, Schienkiewitz A (2013). Fruit and vegetable intake in Germany: Results of the German Health Interview and Examination Survey for Adults (DEGS1). Bundesgesundheitsbl.

[19] Merrill RM, Richardson JS (2009). Validity of Self-Reported Height, Weight, and Body Mass Index: Findings from the National Health and Nutrition Examination Survey, 2001–2006. Prev Chronic Dis.

[20] Neter JE, Dijkstra SC, Visser M, Brouwer IA (2014). Food insecurity among Dutch food bank recipients: a cross-sectional study. BMJ Open.

[21] Neuhauser H, Thamm M, Ellert U (2013). Blood pressure in Germany 2008–2011: Results of the German Health Interview and Examination Survey for Adults (DEGS1). Bundesgesundheitsbl.

[22] OECD (2009). “health status”, in OECD Factbook 2009: Economic, Environmental and Social Statistics.

[23] Pampel FC, Krueger PM, Denney JT (2010). Socioeconomic Disparities in Health Behaviors. Annu Rev Sociol.

[24] Pfeiffer S, Ritter T, Hirseland A (2011). Hunger and nutritional poverty in Germany: quantitative and qualitative empirical insights. Critical public health.

[25] Koch-Institut R (2012). Daten und Fakten: Ergebnisse der Studie “Gesundheit in Deutschland aktuell 2012”. Beiträg zur Gesundheitsberichterstattung des Bundes.

[26] Scheidt-Nave C (2012). German health interview and examination survey for adults (DEGS) - design, objectives and implementation of the first data collection wave. BMC Public Health.

[27] Schneider U, Stilling G, Woltering C (2012). Positive Trends gestoppt, negative Trends beschleunigt. Bericht zur regionalen Armutsentwicklung in Deutschland 2012.

[28] Statista (2015) Europäische Union: Durchschnittsalter der Bevölkerung in den Mitgliedsstaaten im Jahr 2013 (Altersmedian in Jahren). http://de.statista.com/statistik/daten/studie/248994/umfrage/durchschnittsalter-der-bevoelkerung-in-den-eu-laendern/. Accessed 11 June 2015

[29] Statistisches Bundesamt (2010) Alleinerziehende in Deutschland: Ergebnisse des Mikrozensus 2009, Wiesbaden

[30] Statistisches Bundesamt (2015) Verfügbares Einkommen 1991 bis 2012. http://www.vgrdl.de/VGRdL/tbls/tab.asp?rev=RV2011&tbl=tab14&lang=de-DE. Accessed 12 June 2015

[31] Statistisches Bundesamt (2014a) Armut und Soziale Ausgrenzung. Armutsgefährdungsquoten. http://www.amtliche-sozialberichterstattung.de/A1armutsgefaehrdungsquoten.html. Accessed 12 June 2015

[32] Statistisches Bundesamt (2014b) Vorläufige Ergebnisse der Bevölkerungsfortschreibung auf Grundlage des Zensus 2011 (Zensusdaten mit dem Stand vom 10.04.2014) - 2011, Wiesbaden

[33] Su D, Esqueda OA, Li L, Pagan JA (2012). Income inequality and obesity prevalence among OECD countries. J Biosoc Sci.

[34] The Global FoodBanking Network. How Food Banking Works. http://www.foodbanking.org/food-banking/food-banking-works/. Accessed 20 February 2015

[35] Tilak (2006) Has Post-Basic Education any Role in Poverty and Development. Post-Basic Education and Training Working Paper Series – No 7. University of Edinburgh: Centre of African Studies, 2006

[36] Tinnemann P, Pastatter R, Willich SN, Stroebele N (2012). Healthy action against poverty: a descriptive analysis of food redistribution charity clients in Berlin, Germany. Eur J Public Health.

[37] Vucic V, Glibetic M, Novakovic R, Ngo J, Ristic-Medic D, Tepsic J (2009). Dietary assessment methods used for low-income populations in food consumption surveys: a literature review. Br J Nutr.

[38] WHO (2000) Obesity: preventing and managing the global epidemic. WHO Technical Report Series 894, Genf 11234459

[39] Yancey AK, Ortega AN, Kumanyika SK (2006). Effective recruitment and retention of minority research participants. Annu Rev Public Health.

